# Genicular Nerve Radiofrequency Ablation for Persistent Postsurgical Pain After Total Knee Arthroplasty: A Systematic Review of Clinical Effectiveness and Safety

**DOI:** 10.1155/bmri/4650605

**Published:** 2026-04-16

**Authors:** Yerdar Shaukhin, Talgat Anashev, Tamerlan Shokanov

**Affiliations:** ^1^ Department of Traumatology, Research Institute of Traumatology and Orthopedics named after N.D. Batpenov, Karaganda Medical University, Karaganda, Kazakhstan, qmu.kz; ^2^ Department of Orthopedics, Research Institute of Traumatology and Orthopedics named after N.D. Batpenov, Astana, Kazakhstan; ^3^ Department of Orthopedics, Research Institute of Traumatology and Orthopedics named after N.D. Batpenov, Karaganda Medical University, Karaganda, Kazakhstan, qmu.kz

**Keywords:** genicular nerve, knee, osteoarthritis, radiofrequency ablation

## Abstract

**Introduction:**

Total knee arthroplasty is an effective and established surgical treatment of knee osteoarthritis. However, patients report significant perioperative pain and persistent postsurgical pain, which in turn affect the recovery of the patients. Current treatment options include multimodal analgesia. Lately, RFA has been suggested as a procedure for post‐TKA pain management. This systematic review is aimed at critically evaluating the current evidence on the effectiveness and safety of RFA of the genicular nerve as a persistent postoperative pain management in patients after TKA.

**Methods:**

This systematic review focused on RCTs and cohort studies. The search of the literature was done on several databases.

**Results:**

Ten studies, including one RCT and nine retrospective cohort studies with a total of 211 patients, were included. Studies included different RFA techniques and imaging modalities. All studies reported improvement in outcome measures after the procedure according to different scales. The bias assessment revealed moderate‐to‐high risk. The statistical analysis was not done due to heterogeneity of the results.

**Conclusion:**

GNRFA is a promising modality, which reports improvement in outcome measures and absence of adverse effects. It is promising for selected patients but requires RCTs for routine use.

## 1. Background

Total knee arthroplasty (TKA) is a cost‐effective surgical treatment for end‐stage knee osteoarthritis refractory to conservative treatment options with reproducible long‐term outcomes in pain relief and mobility [1, 2]. With an aging population and rising obesity rates, the increasing incidence of knee osteoarthritis has created an increasing demand for TKA. Despite its success, TKA complications include wound infection, instability, malalignment, stiffness, vascular or ligament injury, arthrofibrosis, and pain [3, 4]. Current procedures to minimize the adverse effects and their prophylaxis are wound treatment, strict follow‐up, and evaluation of the prosthesis.

Among these complications, persistent postoperative pain (PPP) remains a significant burden to optimal recovery. This pain affects up to 30% of patients, prolonging recovery and hospital stay, delaying patients′ rehabilitation, affecting satisfaction, and impairing quality of life [5]. Therefore, PPP after TKA is defined as pain that is localized at the operated knee, develops after surgery, and persists more than 6 months after operation while excluding causes such as infection and prosthetic failure [6]. There are currently no prophylaxis measures for persistent postsurgical pain.

The current standard practice of acute pain management is based on a multimodal analgesic approach, which includes different pharmacological and nonpharmacological strategies. Those include drugs (NSAIDS, opioids, and COX‐2 inhibitors), local anesthetics, or peripheral nerve block [7]. However, up to 60% of patients still report severe pain, which means these approaches might be insufficient or not suitable for everyone [8]. Moreover, the effect of current analgesic methods is short term and limited, therefore, repeated procedures might be required [9]. Next, patients might have contraindications to use of some pain management modalities, such as intolerance to opioids and drug‐to‐drug interactions with other medications. This limits the specific approaches of the multimodal analgesia.

In this context, genicular nerve radiofrequency ablation (GNRFA) is an emerging promising technique for pain management. It works by denaturing the sensory nerves delivering pain to the central nervous system (CNS) using conventional, pulsed, or cooled techniques [10]. Although initially it has been successfully used to treat pain in patients with knee osteoarthritis with contraindications to arthroplasties, recent studies suggest this minimally invasive technique can be used for modulating pain after the knee arthroplasty [11]. This area is not studied extensively, and there are limited number of publications analyzing these questions. Currently there is no consensus on efficacy, optimal timing, and approach of RFA in the perioperative period.

Several previous systematic reviews attempted to review this question with varying methodology. Chepalli et al. conducted a systematic review on GNRFA as a treatment of post‐TKA pain; however, this study included case reports and case series, limiting the quality of overall results [6]. Another systematic review by Meiling et al. and Nnake et al. also focused on all publications on treating postsurgical pain using GNRFA, with the search being limited to 2021 and 2022 accordingly [12, 13]. Nnake et al. included studies where GNRFA was conducted before the TKA as a prophylaxis of postsurgical pain [13]. All the studies shared come of the studies included and evaluated the risk of bias as high, therefore mentioning the poor quality of the studies included. However, among these systematic reviews, Nnake et al. evaluated the quality of the studies as good (10/12 for cohort studies and 8/10 for RCT) and risk of bias being low to moderate. It is in contrast to the results of Chepalli et al. and Meiling et al. [6, 12, 13]. Nevertheless, all of the reviews agree on the potential benefits of GNRFA for post‐TKA pain as an efficient adjunct to multimodal analgesia.

This systematic review critically evaluates the use of GNRFA as a PPP management in patients after TKA.

The aim of this review in PICO framework is as follows:

• Population: adults with PPP.
• Intervention: GNRFA (any modality and guidance).
• Comparator: standard multimodal analgesia.
• Outcomes: primary is pain reduction (VAS/NRS scales) and secondary is function (WOMAC/OKOS), opioid use, and adverse events.

By reviewing the original studies, it seeks to determine whether GNRFA can serve as an adjunct to standard analgesia in reducing pain, potentially reducing opioid consumption and hospital length of stay.

To our knowledge, this is the most comprehensive and up‐to‐date systematic review that analyzes higher quality evidence on use of GNRFA in the postoperative period following the TKA. This review follows the PRISMA quality guidelines and includes risk‐of‐bias assessment to ensure reliable synthesis of the available data. The findings are aimed at informing clinical decision‐making and identifying key knowledge gaps for future research.

## 2. Methods

Literature for this systematic review was searched following the methodology reported in the Cochrane Handbook for Systematic Reviews of Interventions. The study is presented according to the PRISMA guidelines; the checklist was filled (in Supplementary Information).

### 2.1. Eligibility Criteria

The inclusion criteria include adults (> 18 years) with persistent postsurgical (post‐TKA) pain or subset within a bigger knee pain cohort. The intervention consisted of GNRFA (thermal, cooled, or pulsed), guided by fluoroscopy or ultrasound, performed for treatment of persistent post‐TKA pain. The study design include randomized controlled trials and cohort studies. Outcomes are presented as quantitative measures of pain and/or function. The minimum follow‐up was > 3 months after GNRFA. Language and availability include full‐text articles published in English. The exclusion criteria comprise case series, case reports, narrative reviews, editorials, surveys, and reviews. Studies of GNRFA for knee osteoarthritis that did not report outcomes or did not indicate inclusion of TKA patients were excluded. Studies evaluating preoperative GNRFA were also excluded.

### 2.2. Literature Search

The search of the literature was done on PubMed, EMBASE, Scopus, Google Scholar, and COCHRANE databases. The last literature search was done on 20th of July 2025. There was no contact with the authors of the articles. The search was limited to publications in the English language. Medical Subject Headings strategy was applied to develop keywords according to the population (knee osteoarthritis), intervention (knee arthroplasty and RFA), and outcome (pain relief and functional improvement). The sample population focused on two groups: those who underwent knee arthroplasty and those who underwent knee arthroplasty and utilized RFA as a perioperative pain management modality.

For PubMed, the complete search strategy were as follows:

(“radiofrequency ablation” [MeSH Terms] OR “radiofrequency ablation” [Title/Abstract] OR “genicular nerve ablation” [Title/Abstract] OR “genicular nerve radiofrequency” [Title/Abstract] OR “RFA” [Title/Abstract]) AND (“arthroplasty, replacement, knee” [MeSH Terms] OR “knee arthroplasty” [Title/Abstract] OR “total knee arthroplasty” [Title/Abstract] OR “TKA” [Title/Abstract]) AND (“pain management” [MeSH Terms] OR “pain relief” [Title/Abstract] OR “postoperative pain” [MeSH Terms] OR “persistent postsurgical pain” [Title/Abstract]).

Search strategies for EMBASE, Scopus, and Cochrane were adapted according to database‐specific subject headings. Google Scholar was used as a supplementary source to identify grey literature and to cross‐check.

### 2.3. Study Selection

All publications according to the search were imported into reference software (EndNote) for organization and to remove duplicates. Two independent reviewers screened all the titles and abstracts to assess their relevance. Publications that were selected as eligible underwent full‐text review by the same reviewers. Differences in choices between reviewers were resolved by discussions. The entire screening was prepared and reflected in the PRISMA 2020 flow diagram.

### 2.4. Data Extraction

Data were extracted using a standardized form that recorded study characteristics, patient demographics, TKA, and GNRFA details (timing after surgery, RFA modality, imaging guidance, and target nerves), comparator, follow‐up duration, pain and functional outcomes, quality‐of‐life measures, opioid consumption, and adverse events. Two reviewers independently extracted data from each study; discrepancies were resolved by discussion or by consulting a third reviewer. When studies included mixed populations, post‐TKA subgroup data were extracted nonchanged, whenever reported. If subgroup‐specific data were not available, aggregate data were used in the tables.

### 2.5. Risk of Bias and Methodological Quality Assessment

Randomized controlled trials and cohort studies were assessed for risk of bias by three independent reviewers using the National Heart, Lung, and Blood Institute (NHLBI) Study Quality Assessment Tools [14]. GRADE assessment was done following GRADE handbook. The results of assessment are presented in Table 1.

**Table 1:** A summary of included articles.

Author, year	Study design	Number of patients	Number of post‐TKA patients treated with RFA	Mean age of patients	RFA type	Imaging guidance	Outcome
Qudsi‐Sinclair et al. 2016 [15]	Double blind RCT	28	14	67+ ‐7	NA	Fluoroscopic	NRS (*p* < 0.001) OKS (*p* < 0.01) KSS (*p* < 0.01) SF‐36 improved PGI‐I improved
Erdem and Sir 2019 [16]	Retrospective cohort	23	6	78+ ‐2.9	Pulsed	Ultrasound	VAS (*p* < 0.01); WOMAC (*p* < 0.01)
Yoshimura et al. 2019 [17]	Retrospective cohort	10	10	NA	NA	Ultrasound	NRS (*p* < 0.05); WOMAC (*p* < 0.05)
Kapural et al. 2019 [18]	Retrospective	183	21	61	Cooled	Fluoroscopic	VAS (*p* < 0.01)
Chen et al. 2020 [19]	Retrospective	265	22	n/a	Thermal	Ultrasound	Positive response 61.1% (95% CI 55.2%–67.0%)
Gönüllü and Tekin 2020 [20]	Retrospective	28	28	67.5	Thermal	Fluoroscopic	VAS (*p* < 0.001); WOMAC (*p* < 0.001)
Belba et al. 2021 [21]	Retrospective	59	46	66.9	Thermal	Ultrasound	NRS (*p* = 0.008) GPE
Khan et al. 2021 [22]	Retrospective	19	19	70.5	Cooled	Fluoroscopic	VAS (*p* < 0.0001); KOOS (*p* < 0.0001)
Shi et al. 2024 [23]	Retrospective	73	27	56.21	Thermal	Fluoroscopic	81.48% success rate (63.3%–91.82% CI) *p* = 0.91 Chi square test
Lo Bianco et al. 2025 [24]	Retrospective observational	50	18	69.7	Thermal	Fluoroscopic	VAS (*p* < 0.0001); WOMAC (*p* < 0.0001); DN4 (*p* < 0.001); EQ‐5D (*p* < 0.01)

The NHLBI Study Quality Assessment Tools for controlled intervention and observational cohort studies were chosen because they provide domain‐specific criteria tailored to nonrandomized designs, which made up most of our evidence base, and are recommended for evaluating clinical intervention studies in these contexts. For each study, three reviewers independently rated all 14 items as “yes,” “no,” or “not reported.” Overall risk‐of‐bias categories were assigned using predefined thresholds:

• Low risk: at least two‐thirds of items rated “yes,” no critical domains (randomization/allocation for RCT; selection, outcome measurement, and confounding for cohorts) rated “no.”
• Moderate risk: between one‐third and two‐thirds of items “yes,” or one critical domain unclear but none clearly high risk.
• High risk: more than one‐third of items rated “no” or at least one critical domain rated “no.” Disagreements were resolved by consensus discussion, and final ratings are summarized in Table 2.

**Table 2:** Risk of bias using study quality assessment tool.

Author, year	1	2	3	4	5	6	7	8	9	10	11	12	13	14	Overall
Qudsi‐Sinclair et al. 2016 [15]	Yes	Yes	Yes	No	n/a	Yes	Yes	Yes	Yes	n/a	Yes	Yes	Yes	Yes	Low
Erdem and Sir 2019 [16]	Yes	Yes	Yes	No	No	Yes	Yes	Yes	Yes	Yes	Yes	No	Yes	No	Moderate
Yoshimura et al. 2019 [17]	Yes	Yes	n/a	Yes	n/a	Yes	n/a	No	Yes	No	Yes	n/a	n/a	No	High
Kapural et al. 2019 [18]	Yes	No	Yes	No	No	Yes	Yes	Yes	Yes	No	Yes	No	n/a	No	High
Chen et al. 2020 [19]	Yes	Yes	Yes	No	No	Yes	Yes	Yes	No	No	Yes	No	Yes	Yes	Moderate
Gönüllü and Tekin 2020 [20]	Yes	Yes	n/a	Yes	n/a	Yes	Yes	No	Yes	No	Yes	n/a	n/a	No	Moderate
Belba et al. 2021 [21]	Yes	Yes	Yes	No	No	Yes	Yes	n/a	Yes	No	Yes	No	Yes	No	Moderate
Khan et al. 2021 [22]	Yes	Yes	Yes	Yes	n/a	Yes	Yes	No	Yes	No	Yes	n/a	n/a	No	Moderate
Shi et al. 2024 [23]	Yes	Yes	n/a	Yes	n/a	Yes	Yes	Yes	Yes	No	Yes	n/a	n/a	Yes	Moderate
Lo Bianco et al. 2025 [24]	Yes	Yes	n/a	Yes	n/a	Yes	Yes	No	Yes	No	Yes	n/a	n/a	No	Moderate

## 3. Results

**Figure 1:**
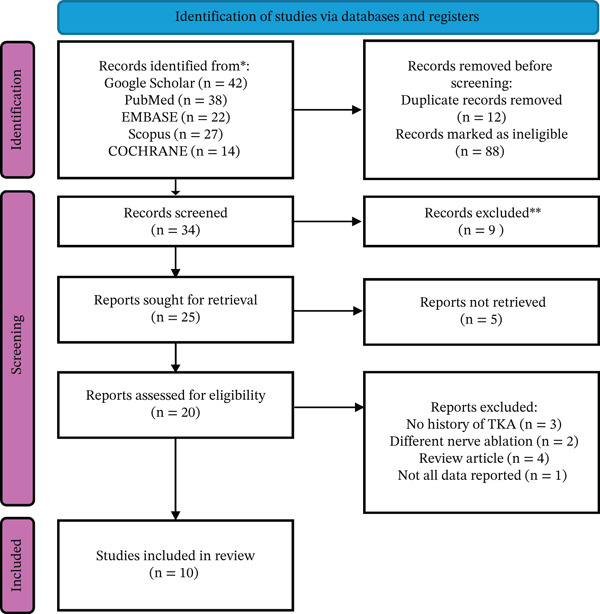
PRISMA flowchart of study selection process.

The study selection process is reflected in the check flowchart in Figure 1. A total of 143 studies were identified from five databases. After removal of duplicates, screening for exclusion criteria, and screening for eligibility, 10 studies were included in this systematic review.

Ten studies (one RCT and nine retrospective cohorts) including 211 patients with persistent post‐TKA pain were included. The mean age of patients ranged from 56.2 to 78 years. The radiofrequency ablation types were different among studies and included thermal (conventional), cooled, and pulsed ones with imaging guidance using fluoroscopy (*n* = 7) and ultrasound (*n* = 3). The summary on the included articles is presented in Table 1.

The overall methodological quality assessment and bias risk assessment of 10 studies was done. Seven of them were marked as moderate, 1 as low bias risk study, and 2 as high bias risk study. The quality assessment summary is represented in Table 2.

The criteria below were assessed [14]:

1. Was the study described as a randomized, a randomized trial, a randomized clinical trial, or an RCT?

2. Was the method of randomization adequate (i.e., use of randomly generated assignment)?

3. Was the treatment allocation concealed (so that assignments could not be predicted)?

4. Were study participants and providers blinded to treatment group assignment?

5. Were the people assessing the outcomes blinded to the participants′ group assignments?

6. Were the groups similar at baseline on important characteristics that could affect outcomes (e.g., demographics, risk factors, comorbid conditions)?

7. Was the overall drop‐out rate from the study at endpoint 20% or lower than the number allocated to treatment?

8. Was the differential drop‐out rate (between treatment groups) at the endpoint 15 percentage points or lower?

9. Was there high adherence to the intervention protocols for each treatment group?

10. Were other interventions avoided or similar in the groups (e.g., similar background treatments)?

11. Were outcomes assessed using valid and reliable measures, implemented consistently across all study participants?

12. Did the authors report that the sample size was sufficiently large to be able to detect a difference in the main outcome between groups with at least 80% power?

13. Were outcomes reported or subgroups analyzed prespecified (i.e., identified before analyses were conducted)?

14. Were all randomized participants analyzed in the group to which they were originally assigned, i.e., did they use an intention‐to‐treat analysis?

Three reviewers independently evaluated quality of the studies. Disagreements were resolved by consensus. Low RoB: 1/10 (RCT blinding/power); Moderate: 7/10(retrospective design); and High: 2/10 (allocation/outcomes unclear). Interrater reliability: kappa = 0.82.

GRADE assessment was conducted according to the handbook, reflecting the evaluation of two types of outcomes: Pain Reduction and function. The assessment revealed low quality due to serious risk of bias, indirectedness and imprecisions, as indicated in Table 3.

**Table 3:** GRADE assessment was conducted following the GRADE Handbook [25].

Outcome	Studies (*n*)	Effect	RoB	Inconsistency	Indirectness	Imprecision	Publication bias	Quality
Pain reduction (VAS/NRS)	Nine (*n* = 211)	Consistent ↓ (*p* < 0.0001 to < 0.05)	Serious	Not serious	Serious	Serious	Undetected	Low
Function (WOMAC/OKS)	Six (*n* = 142)	Consistent ↑	Serious	Not serious	Serious	Serious	Undetected	Low

All 10 studies reported improvements in pain and/or function following GNRFA for persistent post‐TKA pain. Most were uncontrolled retrospective cohorts, so observed changes reflect within‐study trends over time rather than definitive treatment effect.

● Pain outcomes: Nine studies using VAS, NRS, or NPRS reported reductions from baseline to follow‐up (*p* values ranging from < 0.05 to < 0.0001 where tested; e.g., Gönüllü and Tekin; Khan [20, 22])
● Functional outcomes: Six studies reported improvements in WOMAC, KOOS, and OKS scores. Qudsi‐Sinclair et al., the only RCT, showed statistically significant gains in OKS, KSS, and SF‐36, as well as PGI‐I [15].
● Opioid use and satisfaction: One study reported favorable patient global impression of effect (GPE) and reduced medication use [21].
● Neuropathic and quality‐of‐life outcomes: Lo Bianco et al. described improvements in DN4 (neuropathic pain) and EQ‐5D scores (*p* < 0.001 and *p* < 0.01) [24].

No major procedure‐related complications were reported in the included studies; however, adverse events were inconsistently and often briefly documented, so the current evidence is insufficient to draw firm conclusions regarding the safety profile of GNRFA in the post‐TKA population.

### 3.1. Data Synthesis

Due to substantial heterogeneity, no meta‐analysis was performed.

Key sources of heterogeneity included are as follows:

• Clinical: Variable timing of GNRFA post‐TKA (3–24 months), patient characteristics (age 56–78 years, mixed comorbidities), and prior treatments.
• Methodological: One RCT versus nine retrospective cohorts; different comparators (standard care vs. no control); variable follow‐up (1–12 months).
• Outcome/statistical: Diverse scales (VAS/NRS vs. WOMAC/KOOS), timepoints, and analyses (pre–post changes without controls in most studies).

Narrative descriptive synthesis was therefore employed, with GRADE assessment for certainty of evidence.

Despite moderate‐to‐high risk of bias across studies, GNRFA was associated with reported reductions in pain and improvements in function among patients with chronic post‐TKA pain. These descriptive findings suggest potential as an adjunct to multimodal analgesia, but large RCTs are needed to confirm efficacy, optimal timing, and durability relative to alternatives.

## 4. Discussion

This systematic review synthesizes evidence from 10 studies (one RCT and nine cohorts) on GNRFA for persistent post‐TKA pain. Despite consistent reports of pain relief and functional improvements across diverse outcome measures, methodological limitations preclude strong clinical recommendations. The evidence base reveals several strengths alongside critical constraints. All studies described benefit, with the single RCT (Qudsi‐Sinclair 2016) providing the highest quality signal of pain and function gains. Exclusion of case series/poorly reported studies improved rigor over prior reviews. However, small samples, observational designs, variable RFA techniques (thermal/cooled/pulsed), inconsistent timing documentation (3–24 months post‐TKA), and heterogeneous outcomes prevented meta‐analysis and limited causal inference. Inclusion of mixed‐population studies may increase efficacy estimates for post‐TKA subsets, warranting caution in generalization.

### 4.1. Clinical Applicability

GNRFA′s motor‐sparing profile suits patients prioritizing rehabilitation, particularly those refractory to multimodal analgesia. Potential candidates include individuals with opioid intolerance, comorbidities precluding revision surgery, or neuropathic pain features (e.g., positive DN4 screening). Early adoption in high‐risk surgical patients should be avoided pending standardized protocols.

Safety data are reassuring but incomplete. No major complications were documented, yet inconsistent adverse event reporting precludes definitive profiling. Minor events (transient dysesthesias and needle‐site pain) may be underreported given retrospective designs.

Comparison with prior literature contextualizes these findings. Earlier reviews included lower quality evidence and outdated searches, often rating bias as high despite similar efficacy signals [6, 12, 13]. This analysis advances the field through stricter inclusion criteria, updated evidence to July 2025, NHLBI risk‐of‐bias assessment, and GRADE evaluation (low certainty overall).

Future research priorities include large, multicenter RCTs comparing GNRFA against active controls (e.g., repeated blocks and peripheral nerve stimulation), with standardized outcomes (VAS at fixed time points and opioid equivalents), prespecified timing strata, and comprehensive safety monitoring. Cost‐effectiveness analyses and long‐term durability (> 12 months) remain unexplored. Patient‐reported thresholds for meaningful improvement would guide clinical decision‐making.

## 5. Conclusion

GNRFA shows promise as an adjunct for persistent post‐TKA pain, with all included studies reporting improvements in pain and function and no major adverse events documented. However, the evidence, that is dominated by small retrospective cohorts remains low certainty (GRADE), with high risk of bias, heterogeneity and incomplete reporting limiting generalizability. GNRFA may be considered cautiously for carefully selected patients (e.g., refractory to opioids and nonsurgical candidates), with full disclosure of evidential limitations. Large, well‐powered RCTs evaluating standardized protocols, timing, and comparators against alternatives are essential before routine adoption.

## Author Contributions

All authors had full access to the data in the study and take responsibility for the integrity of the data and the accuracy of the data analysis. Conceptualization: Y.S., T.A, and T.S; methodology: T.A.; investigation: Y.S.; formal analysis: Y.S. and T.S.; resources: T.S.; writing—original draft; T.S., T.A., and T.S.; writing—review & editing: Y.S.; visualization: T.A.; supervision: T.S.

## Funding

No funding was received for this manuscript.

## Ethics Statement

The authors have nothing to report.

## Consent

The authors have nothing to report.

## Conflicts of Interest

The authors declare no conflicts of interest.

## Supporting information


**Supporting Information** Additional supporting information can be found online in the Supporting Information section. This systematic review was prepared according to PRISMA Guideline. The checklist below presents a list of points, following which ensures transparency and accuracy of the systematic review.

## Data Availability

Data sharing is not applicable to this article as no datasets were generated or analyzed during the current study.
